# Generation of KS-58 as the first K-Ras(G12D)-inhibitory peptide presenting anti-cancer activity in vivo

**DOI:** 10.1038/s41598-020-78712-5

**Published:** 2020-12-10

**Authors:** Kotaro Sakamoto, Teruaki Masutani, Takatsugu Hirokawa

**Affiliations:** 1 Research and Development Department, Ichimaru Pharcos Company Limited, 318-1 Asagi, Motosu, Gifu 501-0475 Japan; 2 Cellular and Molecular Biotechnology Reseach Institute, National Institute of Advanced Industrial Science and Technology, 2-4-7 Aomi, Koto-ku, Tokyo, 135-0064 Japan; 3 Transborder Medical Research Center, University of Tsukuba, 1-1-1 Tennodai, Tsukuba, 305-8575 Japan; 4 Division of Biomedical Science, University of Tsukuba, 1-1-1 Tennodai, Tsukuba, 305-8575 Japan

**Keywords:** Cancer, Drug discovery

## Abstract

Ras mutations (e.g., occur in K-Ras, N-Ras, and H-Ras) are one of the most desirable and promising drug targets in chemotherapy treatments for cancer. However, there are still no approved drugs directly targeting mutated Ras. In 2017, an artificial cyclic peptide, KRpep-2d, was discovered as the first selective inhibitor of K-Ras(G12D), the most frequent K-Ras mutation. Here, we report the generation of KS-58, a KRpep-2d derivative that is identified as a bicyclic peptide and possess unnatural amino acid structures. Our in vitro data and molecular dynamics simulations suggest that KS-58 enters cells and blocks intracellular Ras–effector protein interactions. KS-58 selectively binds to K-Ras(G12D) and suppresses the in vitro proliferation of the human lung cancer cell line A427 and the human pancreatic cancer cell line PANC-1, both of which express K-Ras(G12D). Moreover, KS-58 exhibits anti-cancer activity when given as an intravenous injection to mice with subcutaneous or orthotropic PANC-1 cell xenografts. The anti-cancer activity is further improved by combination with gemcitabine. To the best of our knowledge, this is the first report of K-Ras(G12D)-selective inhibitory peptide presenting in vivo anti-cancer activity. KS-58 is an attractive lead molecule for the development of novel cancer drugs that target K-Ras(G12D).

## Introduction

Ras has been identified as an intracellular membrane-anchored GTPase and functions as OFF/ON molecular switch that controls cell proliferation signals^1–3^. GDP-bound Ras signals OFF states, while GTP-bound Ras signals ON states. This molecular switch cycle controls the intracellular translation of extracellular signals through the activation of cell surface receptors (e.g., epidermal growth factor receptor or fibroblast growth factor receptors). These two Ras states are supported by guanine nucleotide exchange factors (GEFs), such as Son of Sevenless homolog 1 (SOS1) and GTPase-accelerating proteins (GAPs). GEFs were determined to promote the release of GDP from Ras and the subsequent uptake of GTP. The GTP-bound Ras then interacts with downstream signal molecules, such as B-Raf proto-oncogene serine/threonine kinase (BRAF) and phosphoinositide 3-kinase (PI3K). After signal translation, GTP is hydrolyzed and released from Ras via intrinsic GTPase activity and GAPs.

In cancer cells, single amino acid mutations at multiple positions of Ras, which contains 188–189 amino acid residues, are frequently observed. These mutations attenuate the intrinsic and GAP-mediated GTP hydrolysis of Ras, which results in the enhancement and elongation of cell proliferation signals^1–3^. About 30% of human cancers express mutated Ras, further using the mutation as a major driver of growth. In the Ras family, K-Ras, N-Ras, and H-Ras mutants are attractive drug targets for cancer treatments. K-Ras amino acid mutations are particularly interesting, as they are found in 20% of various human cancers. The three most common mutation positions in K-Ras are at Gly^12^, Gly^13^, and Gln^61^. The order of frequency observed at Gly^12^ is G12D, G12V, G12C, G12A, G12S, and G12R. K-Ras(G12D) is, therefore, one of the most important chemotherapy drug targets. For example, K-Ras(G12D) is very commonly observed in pancreatic cancer, which can be considered a representative of the various intractable cancers.

Despite more than 30 years of research, there are no drugs currently on the market that directly target wild-type (WT) and/or mutated Ras. Since Ras has less druggable pockets for conventional small molecules, other modalities and approaches, such as peptides^4–6^, artificial proteins^7,8^, mimetics of antibody variable fragments^9^, antisense oligonucleotide (e.g., AZD4785)^10^, and targeted covalent inhibitors (e.g., ARS-1620, AMG 510, and MRTX849)^11–14^, have been examined. AZD4785, which targets both WT and mutated K-Ras isoforms, was the first to start the clinical trials, but development was unfortunately discontinued in 2019 after phase I completion of the clinical trial. Two small molecular covalent binders to Cys^12^ of GDP-bound K-Ras(G12C), AMG 510 and MRTX849, are currently in phase II of their clinical trials. Development of the pan-Ras inhibitor BI-1701963, which binds to SOS1 and inhibits the exchange of GDP and GTP in Ras, is also proceeding to phase II of clinical trial^15^. Hence, competition for Ras-targeting drug development is intensifying.

In 2017, Sakamoto et al. reported an artificial 19-mer cyclic peptide KRpep-2d, Ac-RRRR-_c_(CPLYISYDPVC)-RRRR-NH_2_ that selectively binds to both GDP-bound K-Ras(G12D) and GTP-bound K-Ras(G12D) at subnanomolar *K*_D_ values and inhibits the exchange of GDP to GTP in K-Ras(G12D) at an IC_50_ value of 1.6 nM^16–18^. The biophysical characteristics of KRpep-2d were evaluated in detail and have also been established by another research group^19^. KRpep-2d has been identified to have a disulfide bond between two Cys residues that is essential for its peptide cyclic structure and controls its binding and inhibition activities. However, the bond would be cleaved under intracellular reductive conditions. The inhibition activity of KRpep-2d in cell-free enzyme assays is dramatically attenuated in the presence of the reducing regent dithiothreitol (DTT)^16^. The in vitro cell growth suppression activity of KRpep-2d is selective to K-Ras(G12D)-expressing cells^16^.

Here, we report a series of KRpep-2d derivative peptides produced by unnatural amino acid introductions. First, we predicted substitutable amino acid residues in KRpep-2d by using previously reported X-ray crystal structure information^18^. Second, we synthesized a series of single amino acid substitutions and further evaluated their binding activity to recombinant K-Ras(G12D) protein. Third, we combined these unnatural amino acid introductions in each position. Finally, KS-58 was identified as the best derivative in this study because it possessed the strongest cell growth suppression activity and remarkable resistance to protease degradation. Consequently, KS-58 presented in vivo anti-cancer activity when injected intravenously. Here we describe the molecular design and biochemical/biological activities of KS-58.

## Results

### Prediction of substitutable amino acid residues in KRpep-2d

**Figure 1:**
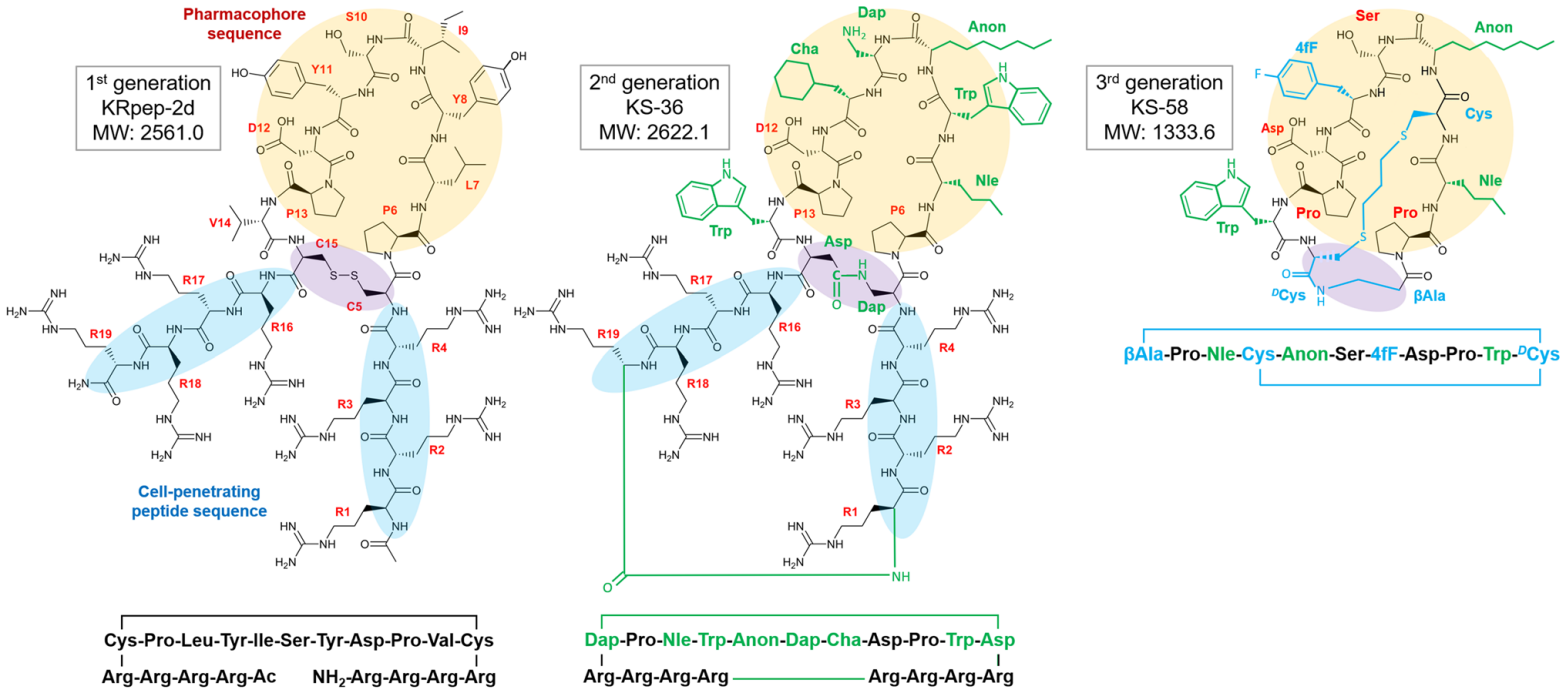
Chemical structures of KRpep-2d, KS-36, and KS-58.

**Figure 2:**
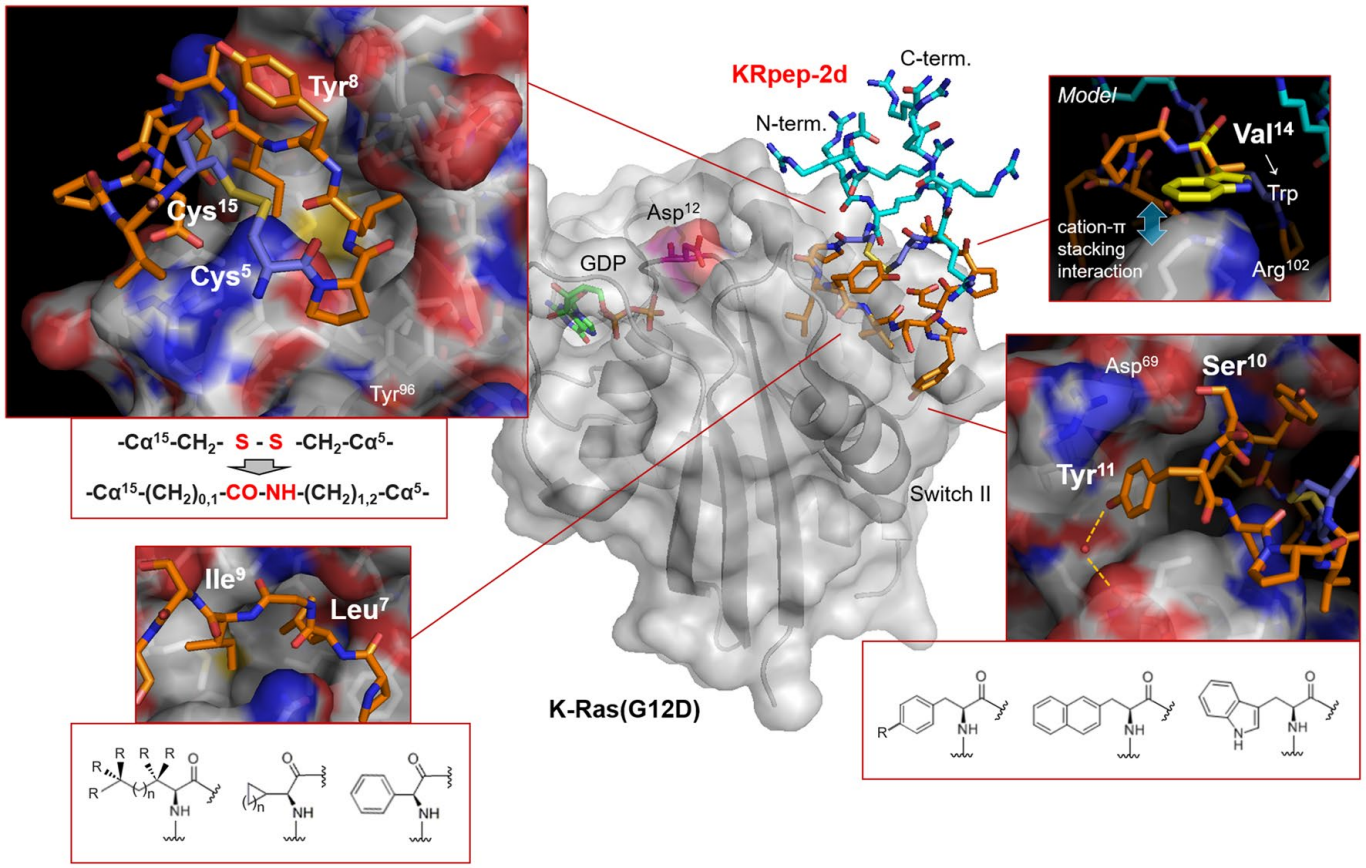
Prediction and design of amino acid substitutions in each amino acid position of KRpep-2d. The complex of ribbon diagram of K-Ras(G12D)^GDP^ and stick model of KRpep-2d (PDB ID: 5XCO) are shown in the center. Purple amino acids are Cys^5^ and Cys^15^. Orange amino acids indicate pharmacophore sequence (Pro^6^–Val^14^). Cyan amino acids present Arg^1–4,16–19^ residues. Chemical structures of representative amino acid examples that were predicted to improve biochemical functions of KRpep-2d in Leu^7^, Ile^9^, and Tyr^11^ are listed. In the upper left side box, Arg residues at both termini of the KRpep-2d were excluded for clarity. The figure was made using PyMOL.

To predict the substitutable amino acid residues in KRpep-2d, X-ray crystal structure information of the K-Ras(G12D)/KRpep-2d complex (PDB ID: 5XCO) was utilized^18^. As shown in Fig. 1, KRpep-2d has certain structural properties as follows: (A) the peptides are cyclized by a disulfide bond between side chains of Cys^5^ and Cys^15^; (B) 6th–14th position amino acids mainly interact with K-Ras(G12D); and (C) 1st–4th and 16th–19th position Arg residues function as a cell-penetrating peptide (CPP). From this information, we devised three strategies: (A) replacing the disulfide bond with an amide bond that has resistance to reductive cleavage; (B) introducing unnatural amino acids to the 6th–14th positions in order to increase the binding affinity to K-Ras(G12D); and (C) connecting the N-terminus amino-group and the C-terminus carboxy-group to each other via an amide bond for main chain cyclization that would improve protease degradation resistance and cell membrane permeability of the peptides^20–23^. Our detailed predictions for each amino acid position are shown in Fig. 2.

### Screening of effective single amino acid substitutions

**Figure 3:**
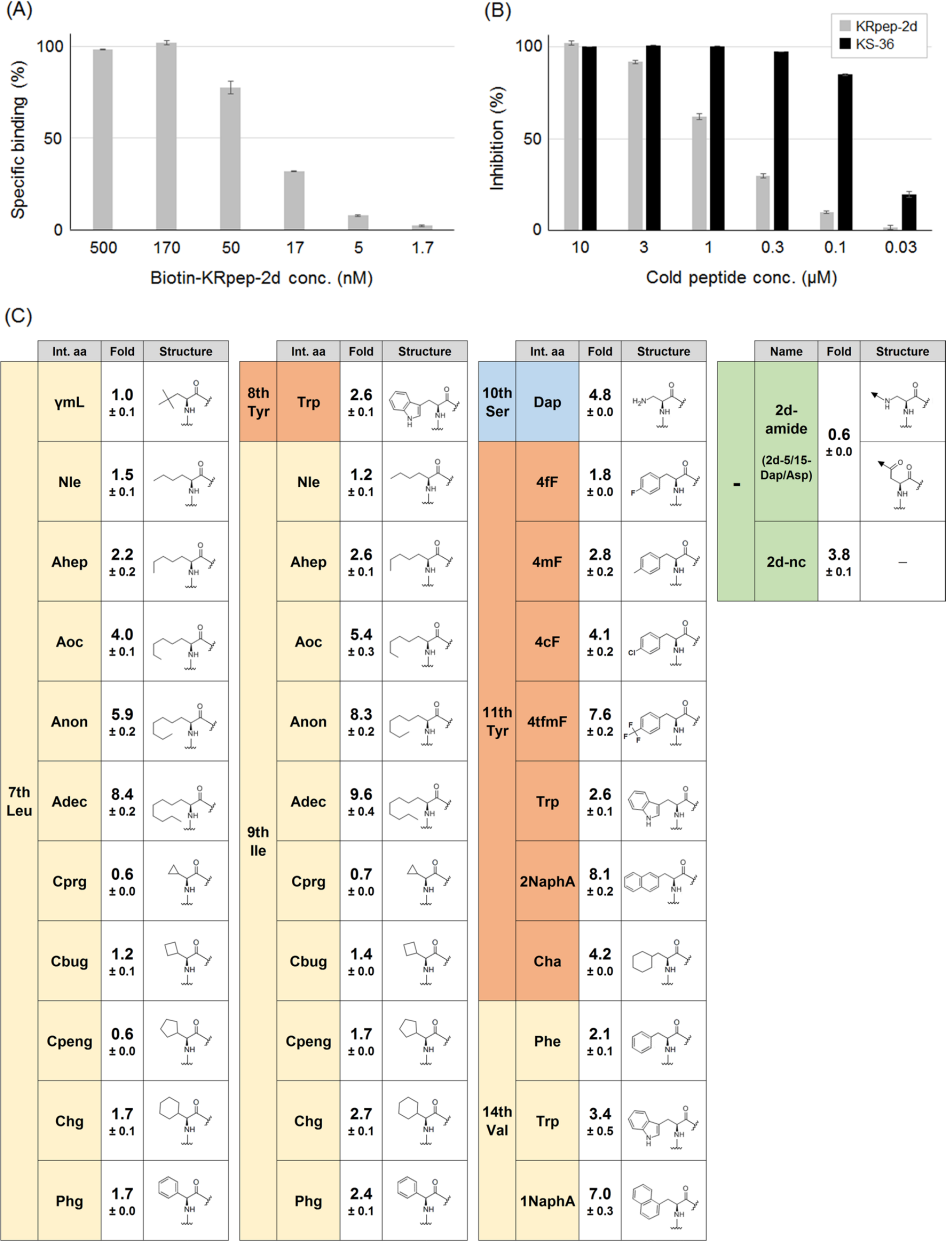
Screening of KRpep-2d derivatives. (**A**) K-Ras(G12D)-binding activity of Biotin-KRpep-2d in ELISA (n = 4, ± SEM). (**B**) Competitive inhibition activities of KRpep-2d and KS-36 against the interaction of plate-coated K-Ras(G12D) and Biotin-KRpep-2d (100 nM) (n = 4, ± SEM). (**C**) Amino acid structures introduced into KRpep-2d derivatives and their K-Ras(G12D)-binding activities. Positions of the substituted amino acids in KRpep-2d and introduced amino acids are listed. K-Ras(G12D)-binding activities are listed as fold-value compared to the parental KRpep-2d set as 1.0 (n = 4, ± SEM). 2d-amide is an amide bond (Dap^5^–Asp^15^) cyclized KRpep-2d, and 2d-nc is main chain cyclized KRpep-2d.

Based on our predictions, 35 peptides were designed, chemically synthesized, and evaluated for their binding activity to recombinant K-Ras(G12D) protein via a competition binding assay. In this assay, K-Ras(G12D)-binding activity of non-labeled peptides could be estimated as a competitive inhibition activity against the binding of biotin-labeled KRpep-2d to K-Ras(G12D). Biotin-KRpep-2d bound to K-Ras(G12D) had an EC_50_ value of 30 nM (Fig. 3A), and its binding was inhibited in the presence of KRpep-2d (Fig. 3B). As listed in Fig. 3C, almost all amino acid substitutions presented stronger inhibition activities than non-labeled, parental KRpep-2d, indicating that predicted amino acid substitutions contributed to the increase of binding activity of these peptides to K-Ras(G12D). For example, introduction of hydrophobic linear aliphatic amino acids to the 7th and 9th positions has provided stronger binding activities in comparison to the introduction of cyclic aliphatic amino acids or the aromatic amino acid, phenylglycine (Phg). The 11th position also seemed to prefer the hydrophobicity increase. The (S)-2,3-diaminopropanoic acid (Dap) introduction to the 10th position has significantly enhanced binding activity by forming a salt bridge binding to Asp^69^ of K-Ras(G12D). The 2d-amide (amide bond cyclized KRpep-2d) showed 0.6-fold weaker binding activity than KRpep-2d, suggesting that amide bond cyclization could be an alternative to disulfide bond cyclization, though the binding activity was slightly attenuated. 2d-nc (main chain cyclized KRpep-2d) has also exhibited 3.8-fold stronger binding activity than KRpep-2d, implying that the main chain cyclization provides stable pharmacophore conformation for K-Ras(G12D)-binding. Thus, predicted amino acid substitutions, including amide bond cyclization and main chain cyclization, were allowed for K-Ras(G12D)-binding of peptides.

### Combination of single amino acid substitutions in each position

We selected a combination of these amino acid substitutions for further research after thorough consideration of their binding activity, lipophilicity, water solubility, and synthesis cost. As a result, bicyclic peptide KS-36, _c_[Arg-Arg-Arg-Arg-_c_(Dap-Pro-Nle-Trp-Anon-Dap-Cha-Asp-Pro-Trp-Asp)-Arg-Arg-Arg-Arg] (main chain cyclization, and also cyclization with side chains of Dap^5^ and Asp^15^) was fully designed and synthesized (Fig. 1). In a competition binding assay, the K-Ras(G12D)-binding activity of KS-36 was estimated to be about 30-fold stronger than that of parental KRpep-2d, but the IC_50_ value could not be accurately evaluated because of the steeply curved Hill slope (Fig. 3B). Next, the proliferation of A427 cells (human lung carcinoma, G12D mutant) in the presence of the peptide was measured using ATP quantification. KS-36 and parental KRpep-2d suppressed A427 cell proliferation down to 33.1% and 56.4% at a peptide concentration of 30 µM, respectively (Table 1).

**Table 1:** Cell proliferation rate of A427 cells in the presence of peptide.

Name	Cell proliferation (%)
KRpep-2d	56.4 ± 9.4
KS-36	33.1 ± 9.6
MC-βAla/Cys	98.3 ± 8.0
BC-βAla/Cys/DIE	75.4 ± 0.8
BC-βAla/Cys/DIP	80.1 ± 1.5
BC-βAla/Cys/DIB	84.7 ± 4.6
MC-βAla/^*D*^Cys	99.1 ± 0.7
BC-βAla/^*D*^Cys/DIE	77.4 ± 4.6
BC-βAla/^*D*^Cys/DIP	72.7 ± 2.5
BC-βAla/^*D*^Cys/DIB	82.4 ± 4.9
MC-γAba/Cys	99.4 ± 3.0
BC-γAba/Cys/DIE	79.0 ± 3.8
BC-γAba/Cys/DIP	84.7 ± 7.6
BC-γAba/Cys/DIB	88.5 ± 4.4
MC-γAba/^*D*^Cys	101.2 ± 5.3
BC-γAba/^*D*^Cys/DIE	86.1 ± 3.7
BC-γAba/^*D*^Cys/DIP	88.8 ± 2.7
BC-γAba/^*D*^Cys/DIB	92.0 ± 3.9
BC-βAla/^*D*^Cys/DIP(Anon^5^)	58.3 ± 3.6
BC-βAla/^*D*^Cys/DIP(Anon^5^/Phe^10^)	27.6 ± 2.8
BC-βAla/^*D*^Cys/DIP(Anon^5^/1NaphA^10^)	34.3 ± 4.1
BC-βAla/^*D*^Cys/DIP(Anon^5^/Trp^10^) = KS-58	21.1 ± 5.5
BC-βAla/^*D*^Cys/DIP(Adec^5^/Trp^10^)	23.4 ± 5.7
BC-βAla/^*D*^Cys/DIP(Anon^3^/Trp^10^)	25.0 ± 3.6
BC-βAla/^*D*^Cys/DIP(Adec^3^/Trp^10^)	28.1 ± 4.2
BC-βAla/^*D*^Cys/DIP(Aoc^3^/Aoc^5^/Trp^10^)	30.3 ± 4.8
BC-βAla/^*D*^Cys/DIP(Anon^3^/Anon^5^/Trp^10^)	33.2 ± 1.3
KS-58(monocyclic)	87.1 ± 1.4
KS-58(4fF7TyrOme)	30.3 ± 5.2
KS-58(4fF7Trp)	55.5 ± 4.6
KS-58(4fF7Cha)	37.7 ± 2.6
KS-58(Ser6Dap)	94.8 ± 9.9

Peptide concentration was 30 µM (n = 4, ± SEM). Peptide name is basically given as MC-X/Z or BC-X/Z/liker, wherein MC means monocyclization, BC means bicyclization, X is the N-terminus first amino acid, and Z is the C-terminus last amino acid of _c_[X-Pro-Nle-_c_(Cys-Ile-Ser-4fF-Asp-Pro-Val-Z)].

### Design of molecular weight reduced bicyclic peptides

Although KS-36 showed cell growth suppression activity, it was found to be weaker than we expected. We hypothesized that the cell membrane permeability of KS-36 is dependent on the CPP region (Arg^1−4,16−19^) and that the CPP cell membrane permeability mechanism would cause its cell growth suppression activity to bottom out, since the mechanism is primarily an energy-dependent endocytosis pathway. Next, we hypothesized that cell membrane permeability of the peptide may be improved by passive diffusion when the CPP region is excluded, because the molecular weight of the peptide is reduced by about 50%. Previous study demonstrated that deletion of the CPP region (Arg^1−4,16−19^) led to significant attenuation of the mutated Ras-binding affinity of KRpep-2d^16,17^. One reason for this may be that the structural conformation of the pharmacophore region (6th–14th position amino acids) is determined to be supported by the CPP region^18^. Since we noticed that there is little distance between Tyr^8^ and Cys^15^ of KRpep-2d (Fig. 2), we designed 12 bicyclic peptides, _c_[X-Pro-Nle-_c_(Cys-Ile-Ser-4fF-Asp-Pro-Val-Z)], where X is βAla (NH_2_–CH_2_–Cα) or γAba (NH_2_–CH_2_–CH_2_–Cα) and Z is Cys or ^*D*^Cys. Stable bicyclic conformation is formed by an amide bond between the main chains of βAla^1^/γAba^1^ and Cys^11^/^*D*^Cys^11^ and a thioether bond through side chains of Cys^4^ and Cys^11^/^*D*^Cys^11^ and halogenated alkyl linkers, DIE (1,2-diiodoethane, I–CH_2_–CH_2_–I), DIP (1,3-diiodopropane, I–CH_2_–CH_2_–CH_2_–I), or DIB (1,4-diiodobutane, I–CH_2_–CH_2_–CH_2_–CH_2_–I). These bicyclizations should be the new support structures as alternatives to the CPP region. As a result of the A427 cell proliferation assay, the optimal combination (X = βAla, Z = ^*D*^Cys, and DIP bridging) was identified (Table 1). Monocyclic peptides, which were cyclized only by main chain amide bonds, displayed weaker cell growth suppression activity than those of bicyclic peptides (Table 1), suggesting that bicyclization was able to provide active conformation of the pharmacophore regions and/or improved cell membrane permeability of the peptides.

Next, we examined amino acid substitutions at Nle^3^, Ile^5^, and Val^10^ of BC-βAla/^*D*^Cys/DIP (Table 1). By introducing Anon to the 5th position and Phe/Trp/1NaphA to the 10th position, which improved cell growth suppression activity. Of them, combination of Anon^5^ and Trp^10^ were deemed most effective. Therefore, other combinations of amino acid substitutions at the 3rd and 5th positions against Trp^10^ were also examined. For example, in the case of Anon^3^, the addition of the combination of Anon^5^ and Trp^10^ did not improve cell growth suppression activity. When 4fF^7^ was substituted to Tyr(Ome), Trp or Cha, cell growth suppression activity was not improved. From the results shown in Table 1, bicyclic peptide KS-58, _c_[βAla-Pro-Nle-_c_(Cys-Anon-Ser-4fF-Asp-Pro-Trp-^*D*^Cys)] (main chain cyclization and thioether bond cyclization with Cα^4^–CH_2_–S–CH_2_–CH_2_–CH_2_–S–CH_2_–Cα^11^), was selected as the best molecule in this study (Fig. 1). The Ser6Dap substitution of KS-58 was determined to be disadvantageous, suggesting that substitution of the side chain hydroxy group with a positively charged polar amino group decreased membrane permeability (Table 1).

KS-58 suppressed growth of A427 cells and PANC-1 cells (human pancreas carcinoma, G12D mutant) down to 21.1% and 50.1% at the peptide concentration of 30 µM, respectively (Table 2). Its cell growth suppression activities against A549 (human lung carcinoma, G12S mutant), H1975 (human lung carcinoma, WT), MIA PaCa-2 (human pancreas carcinoma, G12C mutant), and Capan-1 (human pancreas carcinoma, G12V mutant) cells were significantly weaker than those against K-Ras(G12D)-expressing cells (Table 2). The activities that suppress the phosphorylation of ERK, which is one of the terminal molecules of the Ras-signal cascade, in A427 cells and PANC-1 cells, were also evaluated. KS-58 reduced the phosphorylation of ERK down to 26.0% and 57.6% at a peptide concentration 30 µM, respectively (Table 2). These values of cell proliferation (%) and pERK (%) displayed good correlation.

**Table 2:** Cell proliferation rate and phosphorylation rate of ERK in the presence of peptide.

Origin	Cell line	K-Ras mutation	KS-58		KRpep-2d	
			Cell proliferation (%)	pERK (%)	Cell proliferation (%)	pERK (%)
Lung	A427	G12D	21.1 ± 5.5	26.0 ± 6.0	56.4 ± 9.4	38.5 ± 3.3
	A549	G12S	81.3 ± 1.5	NT	90.8 ± 7.8	NT
	H1975	WT	92.7 ± 0.3	NT	98.0 ± 2.3	NT
Pancreas	PANC-1	G12D	50.1 ± 4.1	57.6 ± 7.6	80.4 ± 4.7	63.9 ± 4.8
	MIA PaCa-2	G12C	86.8 ± 2.3	NT	93.3 ± 3.5	NT
	Capan-1	G12V	94.9 ± 2.2	NT	95.4 ± 4.4	NT

Peptide concentration was 30 µM (n = 4, ± SEM). NT means not tested.

### Ras-binding activity of Biotin-KS-58

**Figure 4:**
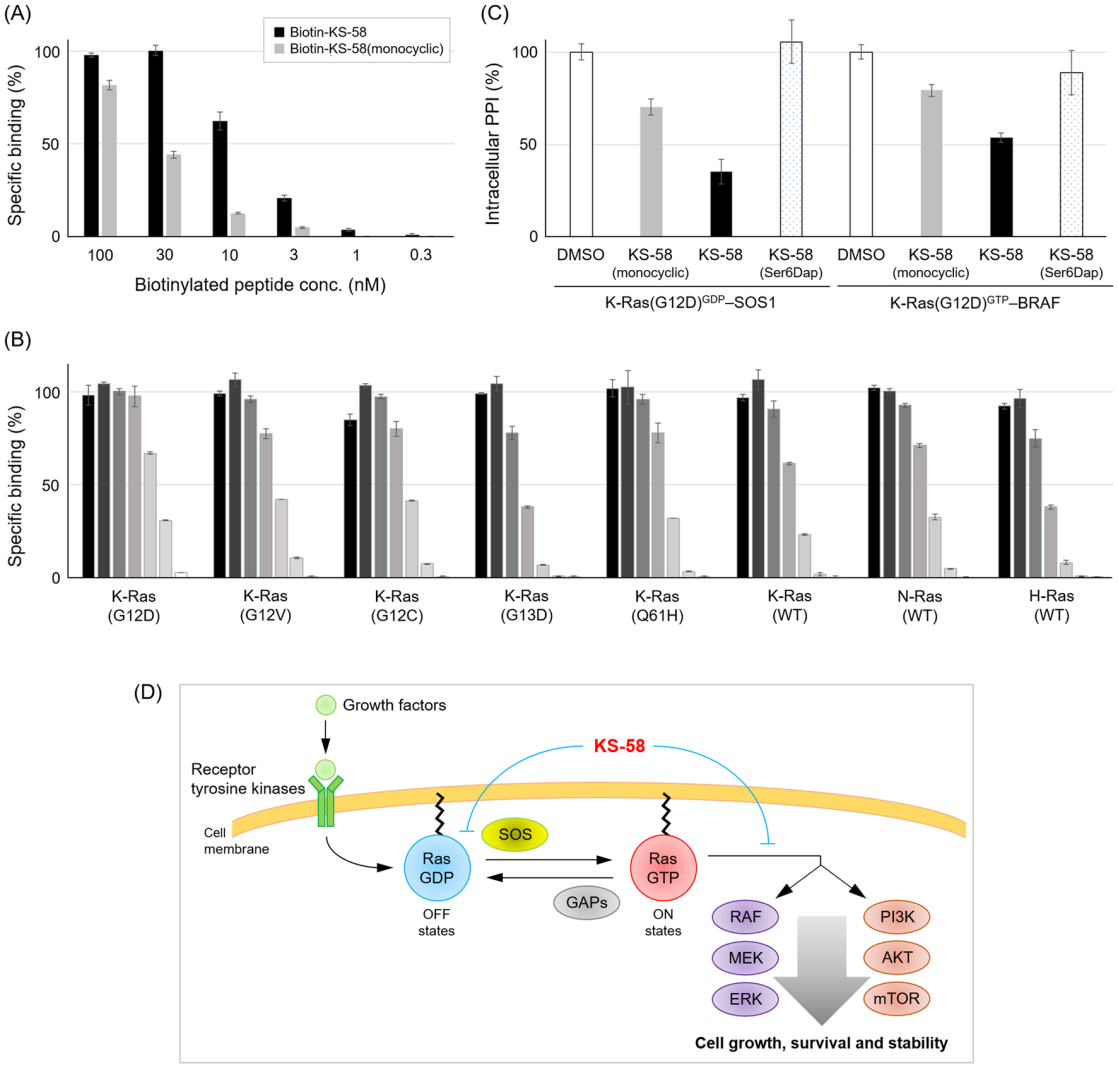
Biochemical characters of KS-58. (**A**) Binding activity of biotinylated peptides to recombinant K-Ras(G12D) protein in ELISA (n = 4, ± SEM). (**B**) Binding activity of different concentrations (from the highest 3 µM and subsequent threefold dilution) of Biotin-KS-58 to recombinant Ras proteins as measured by ELISA (n = 4, ± SEM). (**C**) Inhibition activity of peptides to intracellular Ras–effector proteins interactions. Intracellular PPI in the presence or absence of peptides are detected as luminescence, and the PPI is showed as % value (30 µM, n = 4, ± SEM). (**D**) Proposed K-Ras(G12D)-inhibition mechanism of KS-58.

To confirm the direct binding to Ras, biotin-labeled KS-58, _c_[βAla-Pro-Nle-_c_(Cys-Anon-Ser-4fF-Asp-Lys(εN-biotin)-Trp-^*D*^Cys)] (biotin-labeling to ε-amino group of Lys, main chain cyclization and thioether bond cyclization with Cα^4^–CH_2_–S–CH_2_–CH_2_–CH_2_–S–CH_2_–Cα^11^), was synthesized using Fmoc-Lys(Biot-Acp)-OH (L00835, Watanabe Chemical Industries), and its binding to recombinant K-Ras(G12D) protein was evaluated by ELISA. As shown in Fig. 4A, Biotin-KS-58 demonstrated peptide concentration-dependent binding to K-Ras(G12D) protein. The monocyclic Biotin-KS-58, which has no bridging between Cys^4^ and ^*D*^Cys^11^, revealed weaker binding activity than that of the bicyclic peptide (Fig. 4A). Then, bindings to other recombinant Ras proteins such as K-Ras(G12V), K-Ras(G12C), K-Ras(G13D), K-Ras(Q61H), K-Ras(WT), N-Ras(WT) and H-Ras(WT) were evaluated. As shown in Fig. 4B and Table 3, Biotin-KS-58 exhibited stronger binding to K-Ras(G12D) than to other Ras proteins.

**Table 3:** Binding EC_50_ values of Biotin-KS-58 to Ras proteins.

	K-Ras						N-Ras	H-Ras
	G12D	G12V	G12C	G13D	Q61H	WT	WT	WT
Binding EC_50_ (nM)	22	47	47	150	57	80	60	160
Selectivity (/G12D)	1.0	2.1*	2.1*	7.0*	2.6*	3.6*	2.8*	7.2*

EC_50_ values are listed as average values calculated from Fig. 4B.

*p < 0.05 by Dunnett’s multiple comparison test.

Tables 2 and 3, Fig. 4A,B indicate that the bicyclic peptide KS-58 has inherited K-Ras(G12D)-binding activity and K-Ras(G12D)-mutant cell line selectivity from parental KRpep-2d.

### Inhibition activity of KS-58 against K-Ras(G12D)^GDP^–SOS1 and K-Ras(G12D)^GTP^–BRAF interactions in cytosol

Since KS-58 has inherited the K-Ras(G12D)-binding activity from the parental KRpep-2d, it is expected to bind to both GDP-bound K-Ras(G12D) and GTP-bound K-Ras(G12D) in the same manner as the parent^18^. As a result of these bindings, interactions between Ras and effector proteins, such as SOS1 and BRAF, should be inhibited. To confirm whether KS-58 blocks cytosolic K-Ras(G12D)^GDP^–SOS1 interaction and K-Ras(G12D)^GTP^–BRAF interaction, the NanoBiT system (Promega) was used. In cytosol, the physical association of SmBiT-fused K-Ras(G12D) and LgBiT-fused effector proteins resulted in luminescence. As expected, KS-58 decreased luminescence to 35.5% and 53.8% at a peptide concentration of 30 µM, respectively (Fig. 4C). These values had good correlation with the values of cell proliferation (%) and pERK (%). KS-58(monocyclic) and KS-58(Ser6Dap) showed weaker inhibition activity than KS-58 at the same concentration (Fig. 4C).

The data in Table 2 and Fig. 4A–C suggest that KS-58 enters cells, binds to both forms of intracellular K-Ras(G12D)^GDP/GTP^, and then inhibits K-Ras(G12D)^GDP/GTP^–effector protein interactions, thus preventing downstream Ras signal pathways, such as ERK, and suppressing cell proliferation (Fig. 4C).

### Protease degradation resistance of KS-58

The stability of KS-58 in rat plasma was also evaluated. The peptides were incubated with rat plasma at 37 °C for different time periods (0 and 24 h), and the remaining amount of peptide was then determined using RP-HPLC. By comparing the peak area of the peptide, the remaining amount of peptide after plasma incubation was roughly estimated. As shown in Table 4, KS-58 remained intact for up to at least 24 h, presented good stability under the experimental condition inducing complete degradation of the parental KRpep-2d within 24 h, demonstrating that both unnatural amino acid introduction and bicyclization significantly increased the resistance to protease degradation. Possessing resistance to protease degradation is a very important point for using peptide in vivo.

**Table 4:** Stability of peptide in rat plasma.

		Input (NT)	Incubation time 0 h	Incubation time 24 h
KS-58	Peak area value	491,842	473,575	467,142
	Recovery rate (%)	100	96.3	95.0
	Residual rate (%)	–	100	98.6
KRpep-2d	Peak area value	1,351,046	247,785	Undetectable
	Recovery rate (%)	100	18.3	–
	Residual rate (%)	–	100	–

NT means no-serum treatment.

### Anti-cancer activity of KS-58 in PANC-1 cell xenografts mice

**Figure 5:**
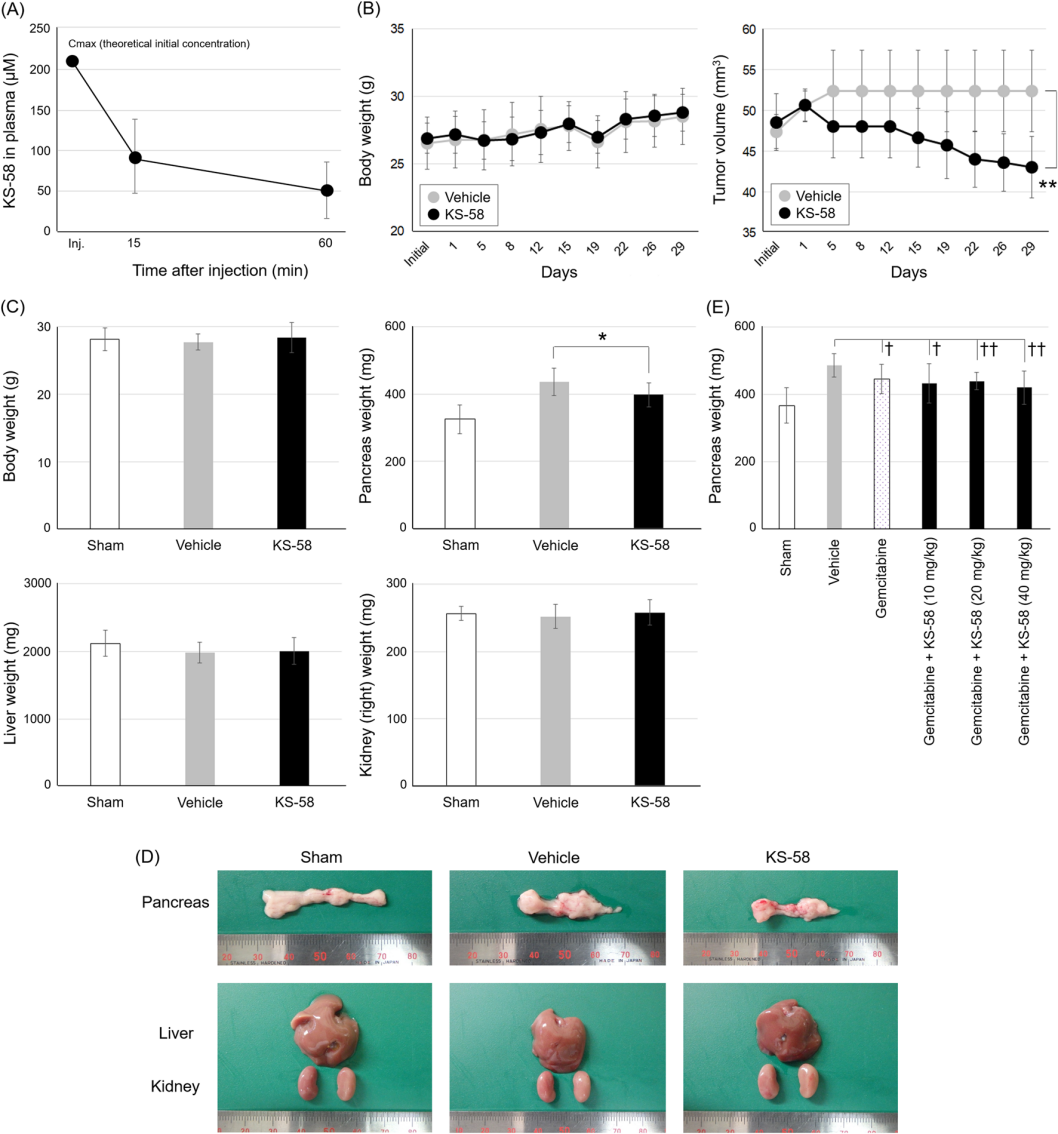
Animal studies using KS-58. (**A**) Pharmacokinetic profile of KS-58 in mice by intravenous injection (20 mg/kg, n = 3, ± SD). C_max_ (theoretical initial concentration) of KS-58 was calculated assuming that the blood volume of mice was 7.3% of body weight. (**B**) In vivo anti-cancer efficacy of KS-58 in mice bearing PANC-1 subcutaneous xenografts (40 mg/kg, n = 4 in vehicle group, n = 10 in KS-58 group). Changes of body weights and tumor volumes are shown (± SD, **p < 0.005 by Student's *t*-test). (**C**) In vivo anti-cancer efficacy of KS-58 in mice bearing PANC-1 orthotopic xenografts (40 mg/kg, n = 7 in each group). Body weight and organ weight (pancreas, liver, and right kidney) are shown (± SD, *p < 0.05 by Student’s *t*-test). (**D**) Representative appearance of each organ of mice in orthotopic xenografts experiments. (**E**) Combination administration test of KS-58 and gemcitabine (n = 10 in each group). Pancreas weight is shown (± SD, †*p* < 0.025 and ††*p* < 0.005 by Williams’ test).

Since KS-58 demonstrated good in vitro data, we proceeded to evaluate it in vivo. Prior to the in vivo anti-cancer activity evaluation, a preliminary pharmacokinetics (PK) study was completed using a 20 mg/kg intravenous injection. Plasma was collected at 15 min, 1 h, 4 h, and 24 h after injection. In general, typical peptides have showed very poor PK profiles, because they seem to be unstable against protease degradation and rapidly cleared from the body by the liver and kidney within a few minutes. KS-58 was detected in plasma 15 min and 1 h after intravenous injection, but it was not detected at 4 h and 24 h, due to the limited detection ability of RP-HPLC. The concentration change indicated a two-compartment model (Fig. 5A). The blood circulation half-life (t_1/2_) in the elimination phase was estimated to be 60 ± 1.9 min. This data indicates that when KS-58 is injected at 20 mg/kg, the peptide concentration in the blood would be retained at more than 30 μM for at least 1 h after the injection, which is the concentration of IC_50_ value KS-58 against PANC-1 cells in the in vitro cell proliferation assay. Since the t_1/2_ of KS-58 was relatively short, we decided to inject at the dose of 40 mg/kg during the pharmacological efficacy test.

K-Ras(G12D) is often observed in pancreatic cancer; therefore, human pancreas carcinoma cell line PANC-1 cells were used for subcutaneous and orthotopic xenograft experiments. Animals were given an intravenous injection of KS-58 (40 mg/kg) once every 2 days for 4 weeks. Results showed significant tumor growth suppression against both xenografts without any adverse side effects, such as loss of body weight or organ swelling (liver and kidney) (Fig. 5B–D). KS-58 reduced the cell growth of PANC-1 to 65%, when the pancreas weight of the Sham group was set to 0% cell growth and the pancreas weight of the vehicle group was set to 100% cell growth in the orthotopic xenograft experiments. This data demonstrates that KS-58 suppresses K-Ras(G12D)-expressing cancer cell growth with reasonable safety features in vivo.

Next, to confirm whether the anti-cancer activity of KS-58 is additive/synergistic in combination with that of any existing drugs, such as gemcitabine, an additional in vivo test was done. When KS-58 treatment (10, 20, 40 mg/kg) was added to gemcitabine treatment (40 mg/kg), the anti-cancer activity was improved in a peptide dose-dependent manner (Fig. 5E) without noticeable adverse side effects (Supplementary Fig. S1). Gemcitabine alone reduced the cell growth of PANC-1 to 66% and KS-58 (40 mg/kg)/gemcitabine combination reduced the cell growth of PANC-1 to 44%, when the pancreas weight of the Sham group was set to 0% cell growth and the pancreas weight of the vehicle group was set to 100% cell growth. This result indicates that co-administration of KS-58 and gemcitabine has an additive effect.

### Molecular dynamics simulations of K-Ras(G12D)^GDP/GTP^-binding modes and lipid membrane permeability of KS-58

**Figure 6:**
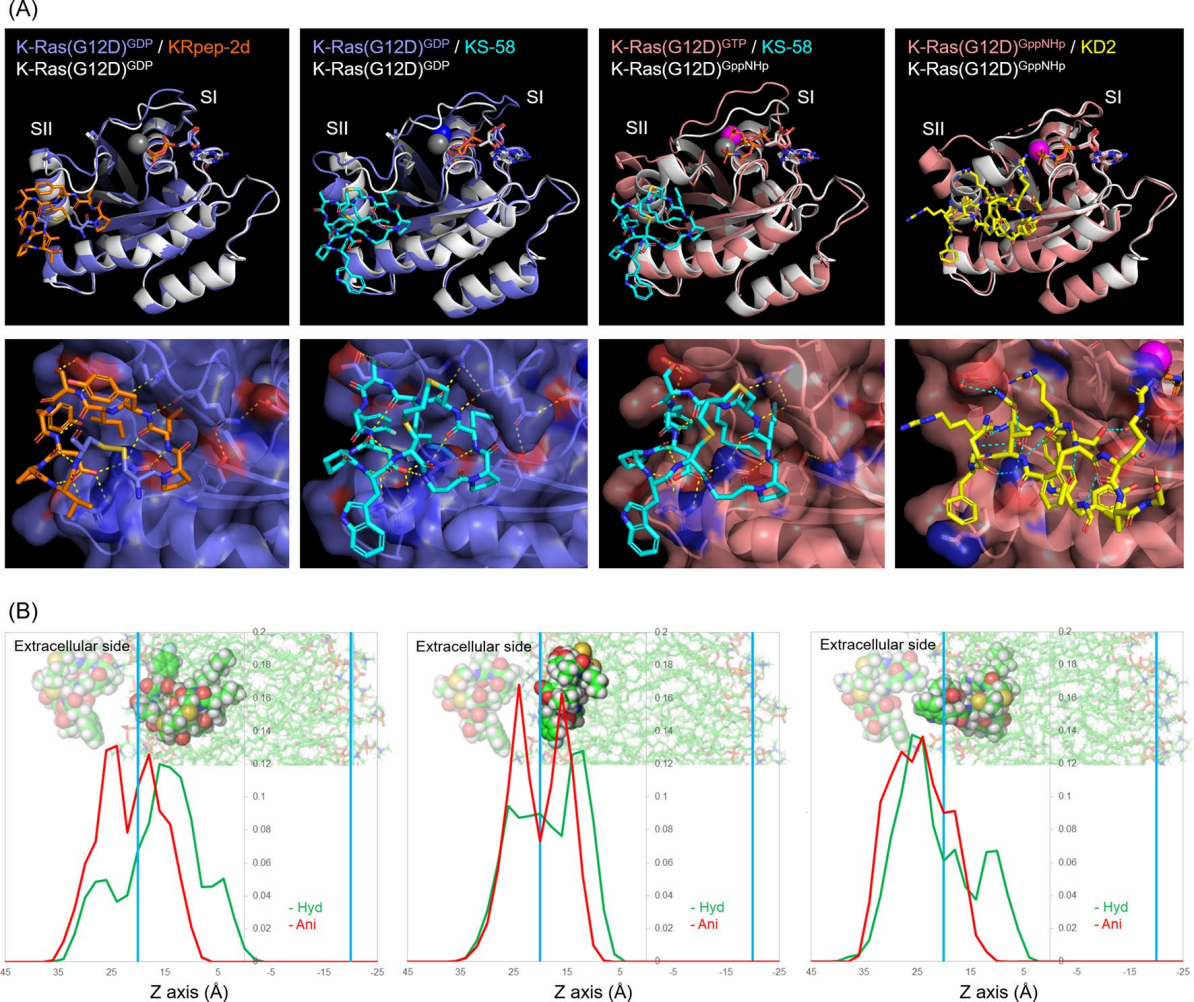
MD simulations of KS-58, KS-58(monocyclic), and KS-58(Ser6Dap). (**A**) Structural comparison of K-Ras(G12D)^GDP^-binding mode and K-Ras(G12D)^GTP^-binding mode of KS-58 (stable snap shots) with K-Ras(G12D)^GDP^ (PDB ID: 4EPR), K-Ras(G12D)^GDP^/KRpep-2d (PDB ID: 5XCO), K-Ras(G12D)^GppNHp^ (PDB ID: 5USJ), and K-Ras(G12D)^GppNHp^/KD2 (PDB ID: 6WGN). The magnesium ions are presented as large spheres. Dash lines indicate hydrogen bonds. Arg residues at both termini of the KRpep-2d were excluded for clarity. (**B**) Lipid membrane accessibility of peptides in MD simulations. Line graph shows moving positions of underlined atoms of Asp^8^ (Cα–CH_2_–COOH, red) and Nle^3^/Anon^5^ (Cα–CH_2_–CH_2_–CH_2_–CH_3_, Cα–CH_2_–CH_2_–CH_2_–CH_2_–CH_2_–CH_2_–CH_3_, green), respectively. KS-58: left panel, KS-58(monocyclic): center panel, KS-58(Ser6Dap): right panel.

To elucidate the target-binding mode and the cell membrane permeability of KS-58, molecular dynamics (MD) simulations were performed. First, predicted binding model of KS-58/K-Ras(G12D)^GDP^ (Fig. 6A) was constructed via computational substitution from the X-ray crystal structure of KRpep-2d/K-Ras(G12D)^GDP^ (PDB ID:5XCO) and MD simulations. Structural frames were taken every 10 picoseconds during the 200 ns production phase. The backbone root-mean-square deviations (RMSDs) of KS-58 from the initial structure after the production phase remained stable at 1.5–4.0 Å (Supplementary Fig. S2A). Therefore, the predicted binding model was considered to be suitable for structure–activity relationship studies. The pharmacophore structure of KS-58 is similar to that of parental KRpep-2d, suggesting that it binds to K-Ras(G12D)^GDP^ in a similar manner as KRpep-2d. The binding free energies of KS-58 and KRpep-2d were calculated as − 106.03 and − 138.69 kcal/mol, respectively. Considering the ligand efficiency (LE: a measurement of the binding energy per heavy atoms of a ligand), KS-58 (LE = – 1.1) had a stronger K-Ras(G12D)^GDP^-binding affinity than KRpep-2d (LE = – 0.77). The key residues that made substantial contributions (ΔG > 10 kcal/mol) to the binding activity were identified. Amino acid substitutions (peptide name is basically given as 2d-X–Z, wherein X means substituted amino acid position in KRpep-2d, Z means introduced amino acid) such as 2d-7-Anon (− 164.23 kcal/mol), 2d-9-Anon (− 166.06 kcal/mol), and 2d-14-Trp (− 156.89 kcal/mol) showed significantly lower binding free energy than parental KRpep-2d (− 138.69 kcal/mol) (Supplementary Fig. S3). This data has good correlation with the results of Fig. 3. Namely, hydrophobic interactions of Anon^5^ and cation–π stacking interaction of Trp^10^ to Arg^102^ were considered to have contributed mainly to the improvement of the binding affinity of KS-58. Using the same method, the binding free energies of the KS-58(monocyclic) and KS-58(Ser6Dap) were also calculated as − 101.01 and − 110.75 kcal/mol, respectively (Supplementary Fig. S2B). The KS-58(monocyclic) results displayed a comparable binding free energy with KS-58, which seems to be inconsistent with the results in Fig. 4A, but is in fact reasonable, since our MD simulations started from an already bound-state, and could not take into account the entropy loss that accompanied the structural peptide change for K-Ras(G12D)-binding. Both monocyclic and bicyclic peptides appear stable after binding.

The data of Fig. 4 indicate that KS-58 has favorable binding profiles (i.e*.* not only selective-binding to K-Ras(G12D) but also cross-binding to its GDP- and GTP-bound forms) as same as parental KRpep-2d. It has been known that the structures of GDP- and GTP-bound forms are different regardless of the WT and the mutants Ras. Especially the conformations of Switch I (SI) (Asp^30^–Tyr^40^) and Switch II (SII) (Thr^58^–Met^72^) are different^24^. SI and SII are involved in binding to effector proteins such as SOS1 and BRAF for signal transduction^24^. The structure of Ras is flexible and changes dynamically to play a role as a molecular switch. Very recently, K-Ras(G12D)^GTP^-selective binding peptide KD2 has been reported^25^. Interestingly, the X-ray crystal structure of KD2/K-Ras(G12D)^GppNHp^ complex (PDB ID: 6WGN) reveals that KD2 binds to the same site as KRpep-2d and the SII conformation is similar to that of KRpep-2d/K-Ras(G12D)^GDP^ complex (PDB ID: 5XCO) (Fig. 6A). We hypothesize that binding modes of KRpep-2d and KS-58 against K-Ras(G12D)^GTP^ are likely similar to those against K-Ras(G12D)^GDP^. As shown in Fig. 6A, binding between KS-58 and K-Ras(G12D)^GTP^ was computationally simulated. Structural frames were taken every 10 picoseconds during the 200 ns production phase. The backbone RMSDs were stable in simulations (Supplementary Fig. S2A). The calculated binding free energy is − 111.02 kcal/mol and comparable to that against K-Ras(G12D)^GDP^ (− 106.03 kcal/mol). Our MD simulations support KS-58’s cross-binding activity to GDP- and GTP-bound forms of K-Ras(G12D).

Next, the lipid membrane permeability of KS-58, KS-58(monocyclic), and KS-58(Ser6Dap) were simulated (Fig. 6B). Unfortunately, they could not penetrate the lipid bilayer in 100 ns of MD simulations, but moved inside. KS-58 showed the deepest invasion degree into the lipid bilayer. When tracing the positions of the side chain of Asp^8^ (anion) and the side chains of Nle^3^/Anon^5^ (hydrophobic), respectively, hydrophobic side chains entered deeper into the lipid membrane, suggesting that KS-58 accesses the lipid membrane in the same direction as amphipathic lipids. The distribution of the positions of these side chains in KS-58(monocyclic) and KS-58(Ser6Dap) overlapped, suggesting that these peptides do not behave like amphipathic molecules and indicating that their lipid membrane accessibility is limited.

These MD simulations provide reasonability to the results of cell growth suppression activity (Table 1) and intracellular PPI inhibition activity (Fig. 4C) of KS-58, KS-58(monocyclic), and KS-58(Ser6Dap). The activity differences between KS-58 and KS-58(monocyclic) would be caused by K-Ras(G12D)-binding activities included entropy loss, and between KS-58 and KS-58(Ser6Dap) would be caused by lipid membrane accessibility.

## Discussion

Development of a drug that directly targets mutated Ras, especially mutated K-Ras, is one of the goals of chemotherapy research in cancer treatments^26^. In order to achieve this, a lot of research in academia and industry has been devoted to this goal for over 30 years. After a number of failures, the targeted covalent inhibitors, AMG 510 and MRTX849, are expected to be used in drugs against K-Ras(G12C). There has been no report, however, of an effective drug candidate in clinical study against K-Ras(G12D), identified as the major K-Ras mutant. Although KS-58 is at an earlier stage than these two molecules, it has potential to be an attractive lead molecule for drug design against K-Ras(G12D). Drug developments using different modalities will increase the success rate that humanity can have against Ras-mutated cancers.

The most innovative point of this study is the generation of a peptide molecule that selectively suppresses K-Ras(G12D)-expressing cell line growth and blocks K-Ras(G12D) functions using two approaches (Fig. 4D), the inhibition of Ras^GDP^–SOS1 interaction (namely, GDP–GTP exchange on Ras) and Ras^GTP^–BRAF interaction. It was also revealed that the peptide molecule KS-58 designed in this study possesses in vivo anti-cancer efficacy. Furthermore, our in vitro data and MD simulations suggested that KS-58 accesses lipid membrane and likely enters cells, despite its molecular weight (MW) of 1333.6 g/mol.

A number of peptide therapeutics with MW > 1000 g/mol, which is beyond Lipinski’s rule of 5 (bRo5) category^27^, have been marketed, but all of them reportedly target extracellular molecules, except for cyclosporine A (CsA, MW = 1202.6 g/mol)^28^. CsA was discovered from *Tolypocladium inflatum* in 1969 and was later approved as an immunosuppressant by the FDA in 1983. It is a monocyclic peptide possessing a non-polar surface and a highly N-methylated backbone. It also displays a membrane permeability via passive diffusion with peptide structure changes^29^. Even in 2020, more than 30 years after CsA approval, designing a molecule such as CsA remains to be difficult, because the detailed mechanisms for providing membrane permeability to bRo5 molecules (especially > 1000 g/mol) by passive diffusion are yet to be fully understood. One feature of CsA is its molecular chameleon property; the peptide structure in the water phase and the lipid phase are different^29^.

Our MD simulations (Fig. 6) suggest that KS-58 accesses lipid membrane, but its molecular structure is rigid, unlike CsA, by the bicyclization. KS-58(monocyclic) seems to have structural flexibility similar to CsA; however, KS-58 showed stronger cell growth suppression activity than KS-58(monocyclic). This result indicates that it is difficult to achieve both target-binding activity and membrane accessibility/permeability in the molecular design of bRo5 by following one precedent, such as CsA. Trial-and-error methods are needed.

Unlike CsA, KS-58 has a negative charged polar residue, Asp^8^, which is critical to K-Ras(G12D)-binding activity, and makes the peptide water soluble. The charged residues make it difficult for peptides to enter cell membranes. KS-58 showed anti-cancer activity in vivo. Since tumor tissues are identified to have acidic conditions (pH = 5.5 − 6.5), the side chain of Asp^8^ (pKa = 4) would become partially deionized, resulting in a potential increase to the lipid membrane accessibility of KS-58. This might be one reason why KS-58 presented anti-cancer activity in vivo, despite the moderate (micromole order) cell growth suppression activity in vitro. In addition, t_1/2_ of KS-58 (60 min) is considered to be an important factor. Asp^8^ and Anon^5^ may cooperatively play a role in the relative extension of t_1/2_ by binding to a basic protein albumin, which absorbs acidic/hydrophobic molecules in blood^30^.

An important question is whether there is any room for further improvement of the KS-58 structure. MD simulations showed K-Ras(G12D)^GDP/GTP^-binding modes of KS-58. All amino acid residues of _c_[βAla-Pro-Nle-_c_(Cys-Anon-Ser-4fF-Asp-Pro-Trp-^*D*^Cys)] would play a clear role to each other to target the allosteric site of K-Ras(G12D). βAla^1^, Cys^4^, and ^*D*^Cys^11^ have been considered essential to form the rigid bicyclic structure of peptide by an amide bond and a thioether bond, which lead to binding activity and are responsible for creating a remarkable resistance against protease degradation of the peptide. As KS-58 presented anti-cancer activity in vivo, improvement of resistance to protease degradation is one of the important points, while cell growth suppression activity of KS-58 was not improved in order of magnitude in compared with that of KRpep-2d. The side chains of Pro^2^, Nle^3^, Anon^5^, and 4fF^7^ interact hydrophobically with K-Ras(G12D). Particularly, the long alkyl chain of Anon^5^ bites into the hydrophobic deep groove of K-Ras(G12D). The long alkyl chain also contributes to the lipid membrane accessibility of the peptide^31–33^. Ser^6^ reportedly interacts via hydrogen bonds with Asp^69^ of K-Ras(G12D). Asp^8^ has been determined to form a critical ion-bond with Arg^102^ of K-Ras(G12D). Two β-turns formed by Ser^6^–4fF^7^ and Pro^9^–Trp^10^ determine the orientation of the side chain of Asp^8^, and the aromatic side chain of Trp^10^ interacts with Arg^102^ of K-Ras(G12D) via a cation–π stacking interaction. Taken together, we concluded that KS-58 is the best peptide molecule to target this allosteric site of K-Ras(G12D) at the present stage and that deletion/substitution of amino acid residues or side chains will be disadvantageous to K-Ras(G12D)-binding. Points to be improved on are the PK profile and the high dose needed. The t_1/2_ of KS-58 is 60 min, which is relatively short, and the dose needed in the in vivo study was 40 mg/kg. Both of these variables will need to be improved using formulation technology. A combination of a drug delivery system into cells such as liposomes or nanoparticles may be an effective method to extend t_1/2_ and decrease the required dose.

As mentioned above, drug developments using different modalities would be effective to overcome Ras-mutated cancer. The first human trial was started in 2018 using AstraZeneca’s antisense oligonucleotide AZD4785, which shows strong protein knockdown activity, but is not selective to mutated K-Ras. Although AstraZeneca reported that WT K-Ras knockdown did not induce any undesirable side effects in animal experiments using mice and monkeys^10^, it has been unclear what adverse effects occur with long-term human use. The detailed reasons have not been provided yet, but unfortunately, the development of AZ4785 was discontinued in 2019.

The mutant Ras-selectivity possessed by AMG 510, MRTX849, and KS-58 would likely be an important factor of drug development against Ras-mutated cancer. Meanwhile, very recently, resistance mechanisms against ARS-1620, which covalently binds to K-Ras(G12C)^GDP^ and inhibits its function, were reported^34^. This study suggests that GTP binds to a newly produced K-Ras(G12C) before ARS-1620 binds, resulting K-Ras(G12C) functions even in the presence of ARS-1620. The fact that KS-58 inhibits both GDP-bound and GTP-bound K-Ras(G12D) may be advantageous in drug development against Ras-mutated cancer.

There are still many areas open to further research, including investigations on anti-cancer effect by combination with other inhibitors to targets of Ras-signal cascade such as BRAF and MEK. At present stage, most importantly, we have successfully generated KS-58, which is the first K-Ras(G12D)-selective inhibitory peptide presenting anti-cancer activity in vivo. KS-58 has been determined to have the potential to begin a new chapter in the chemotherapy treatment of Ras-mutated cancers.

## Materials and methods

### Synthetic peptides

All synthetic peptides were synthesized at SCRUM Inc. (Tokyo, Japan) using Fmoc-based solid-phase peptide synthesis, followed by reverse-phase high performance liquid chromatography (RP-HPLC) purification. Peptide purity was ascertained using analytical RP-HPLC, and structure assignment was performed by MALDI-TOF MS. Disulfide bond formation and thioether bond formation via halogenated chemical linkers were performed according to previous reports, respectively^17,35^. All analytical data of peptides in this report are presented in Supplementary Table S1.

The following briefly describes the synthesis method of KS-58. After synthesis and purification of side chain-protected linear peptides, it was dissolved in DCM, and then, it was mixed with HOAt/EDC and was further dissolved in DMF to activate the C-terminus carboxy-group for amide bond formation with the N-terminus amide-group. The reaction was done on ice for 1 h and further overnight at room temperature. Purified water was added to the solution, which resulted in the recovery of an organic solvent phase. This procedure was repeated for washing. After drying the obtained solution, side-chain deprotection and subsequent ether precipitation were carried out, and the peptide was purified by RP-HPLC using SunFire C18 column (Waters Co, MA, USA). The fraction containing the product was then collected and lyophilized to give main chain-cyclized and side chain-deprotected peptide of KS-58(monocyclic), _c_(βAla-Pro-Nle-Cys-Anon-Ser-4fF-Asp-Pro-Trp-^*D*^Cys) as a white powder with a mass spectrum of [M + H]^+^ 1293.307 (Calc 1293.6), a purity of 95.54%, and an elution time on RP-HPLC (flow rate 1 mL/min) of 14.177 min in linear density gradient elution conditions (A/B = 80/20 − 10/90 for 20 min using 0.1% TFA in water as eluent A and 0.1% TFA in acetonitrile as eluent B). The peptide was dissolved in DMSO and was then mixed with DIP/TCEP dissolved in 100 mM NaHCO_3_/acetonitrile to link side chains of Cys^4^/^*D*^Cys^11^ by chemical linker DIP. The reaction was carried out at 80 °C, and the peptide was purified by RP-HPLC using a SunFire C18 column. The fraction containing the product was collected and lyophilized to obtain bicyclic peptide KS-58, _c_[βAla-Pro-Nle-_c_(Cys-Anon-Ser-4fF-Asp-Pro-Trp-^*D*^Cys)] (main chain cyclization and thioether bond cyclization with Cα^4^-CH_2_-S-CH_2_-CH_2_-CH_2_-S-CH_2_-Cα^11^) as a white powder whose mass spectrum was [M + H]^+^ 1333.620 (Calc 1333.6), with a purity of 95.32% and an elution time on RP-HPLC (flow rate 1 mL/min) of 13.787 min in linear density gradient elution conditions (A/B = 80/20 − 10/90 for 20 min using 0.1% TFA in water as eluent A and 0.1% TFA in acetonitrile as eluent B).

### Cell-free ELISA

Recombinant human K-Ras(G12D) (ab268712), K-Ras(G12V) (ab268713), K-Ras(G12C) (ab268711), K-Ras(G13D) (ab271580), K-Ras(Q61H) (ab96817), K-Ras(WT) (ab156968), N-Ras(WT) (ab268821), and H-Ras(WT) (ab93949) proteins were purchased from Abcam (Cambridge, UK). Ras protein was directly coated to wells of Nunc MaxiSorp microplate in PBS containing 0.01 mM GDP and 10 mM MgCl_2_ overnight at 4 °C. The wells were blocked in PBS containing 0.1% bovine serum albumin (BSA) for 0.5 h at 25 °C.

For binding assays of biotin-labeled peptides, Ras protein (25 μg/mL) was coated to wells of Nunc MaxiSorp 96-well clear plate (439,454) as described above. After washing the wells three times using PBS containing 0.1% Tween20 (PBST), biotin-labeled peptides in PBS containing 0.025% BSA, 0.01 mM GDP, and 10 mM MgCl_2_ were added to the wells. After 0.5 h incubation at 25 °C, the wells were washed with PBST three times. Bound biotin-labeled peptides were detected using horseradish peroxidase (HRP)-conjugated streptavidin (SA) (ab7403, Abcam) and 1-Step Ultra TMB-ELISA Substrate Solution (34028, Thermo Fisher Scientific). The amounts of HRP in the wells were measured by absorbance to 450 nm.

For competition binding assays, Ras protein (250 ng/mL) was coated to wells of Nunc MaxiSorp 96-well clear plate (439454) as described above. After washing the wells three times with PBST, biotin-labeled KRpep-2d (100 nM) in PBS containing 0.025% BSA, 0.01 mM GDP, and 10 mM MgCl_2_ was added to the wells in the presence or absence of non-labeled peptides. After 0.5 h incubation at 25 °C, the wells were washed with PBST three times, and bound Biotin-KRpep-2d was detected using HRP-SA and 1-Step Ultra TMB-ELISA Substrate Solution. The amounts of HRP in the wells were measured by absorbance at 450 nm. Percent inhibition was calculated using the absorbance values from wells without non-labeled peptides as 0% inhibition and values from wells without Ras protein coating as 100% inhibition. IC_50_ values were estimated by equation: IC_50_ = 10^[Log(A/B) × (50 – D)/(C – D) + Log(B)], where A is concentration at > 50% inhibition, B is concentration at < 50% inhibition, C is inhibition rate at concertation A, and D is inhibition rate at concertation B.

### In vitro cell-based assays (cell proliferation assay, Erk-phosphorylation assay, and intracellular PPI assay using NanoBiT)

A427 (ATCC HTB-53), A549 (ATCC CCL-185), H1975 (ATCC CRL-5908), PANC-1 (ATCC CRL-1469), MIA PaCa-2 (ATCC CRL-1420), and Capan-1 (ATCC HTB-79) cells were purchased from ATCC, and each cell was cultured in medium supplemented with 10% fetal bovine serum (FBS), according to the protocol recommended by the manufacture.

The effect of peptides on cell proliferation of A427, A549, H1975, PANC-1, MIA PaCa-2, and Capan-1 cells was determined by 72 h exposure to peptides. Cells were seeded at 1000 cells/well in 96-well tissue culture plates in each growth media and allowed to adhere overnight on day 0. Peptides were solved in DMSO and then diluted with each growth media for a constant final DMSO concentration of 0.5%. Media in the wells were removed and replaced with diluted peptide solutions, and cells were incubated for 3 days. During the incubation, medium containing the peptides was replaced every day. Relative cell numbers were estimated using CellTiter-Glo assay kit (G7570, Promega, WI, USA) according to the manufacturer’s instructions. Luminescence was detected using SpectraMax i3x (MOLECULAR DEVICES, CA, USA). Cell proliferation rate (%) was calculated based on cell numbers at day 0 as 0% and at day 3 without peptide treatment as 100%.

For evaluation of the effect of peptides on Ras–ERK pathways, phosphorylation of ERK was measured by Phospho-ERK1 (T202/Y204)/ERK2 (T185/Y187) DuoSet IC ELISA (DYC1018B-2, R&D SYSTEMS, MN, USA) according to the protocol supplied by the manufacturer. After 16 h serum starvation, cells were treated with peptides diluted in FBS-free medium for 1 h, and then treated with peptides diluted in FBS-containing medium for an additional 1 h. Phosphorylation rate of ERK (%) was calculated based on no serum stimulation as 0% and serum stimulation without peptide treatment as 100%.

Intracellular protein–protein interaction (PPI) inhibition assays were carried out using the NanoBiT system (Promega). Three kinds of plasmids with CMV promoters, which encodes human K-Ras(G12D) fusing SmBiT to the N-terminus, encodes human SOS1 fusing LgBiT to the C-terminus, and encodes human BRAF fusing LgBiT to the C-terminus, were prepared by Promega. Each combination of plasmids was transfected to HEK293T cells by FuGENE HD Reagent (E2312, Thermo Fisher Scientific). After 30 h incubation at 37 °C under 5% CO_2_, the cells were recovered, and seeded to 96-well tissue culture white plate with 10% FBS containing DMEM, and incubated overnight. Peptides were added to the plate and incubated for another 2 h. Nano-Glo (Promega, N2011) was directly added to the plate, and luminescence was immediately monitored. Intracellular K-Ras(G12D)^GDP^–SOS1 and K-Ras(G12D)^GTP^–BRAF interactions (%) were calculated based on no peptide treatment as 100% and only transfection of plasmid encoding LgBiT fusion protein as 0%.

### Evaluation of peptides stability in rat plasma

Each peptide (10 mM, 1 μL) was incubated with rat plasma (20 μL) prepared in house. Immediately after mixing with serum and after 24 h incubation at 37 °C, 80% methanol (200 μL) was added to extract KRpep-2d, and acetonitrile (200 μL) was added to extract KS-58. The mixture was stored for 10 min at 4 °C and centrifuged at 15,000 rpm for 10 min at 4 °C. The supernatant was recovered and was directly supplied to RP-HPLC to determine the remaining amount of unmodified peptides in the sample.

### Pharmacokinetics (PK) studies of KS-58 in mice

Animal experiments were carried out at UNITECH Co. Ltd. (Chiba, Japan) in accordance with the guidelines of the Animal Care and Use Committee of UNITECH (approval No. AGR IMF-190807A-30). KS-58 was dissolved in DMSO and then tenfold diluted by saline. The peptide solution was injected intravenously to Slc:ICR male mice (8 weeks old; Japan SLC, Inc.) (20 mg/kg, n = 3). Blood samples were collected from the mice at 15 min, 1 h, 4 h, and 24 h after injection. The blood was chilled on ice and then centrifuged at 12,000 rpm for 3 min at 4 °C. Plasma protein was precipitated by acetonitrile, and the supernatant was recovered. The remaining peptides in prepared samples were analyzed by RP-HPLC as described above.

### Evaluation of pharmacological efficacy of KS-58 in vivo

Animal experiments were carried out at UNITECH Co. Ltd. in accordance with the guidelines of the Animal Care and Use Committee of UNITECH (approval No. AGR IMF-190807A-31, -33, AGR IMF-20615A-30). To establish subcutaneous xenograft tumor models, 1 × 10^7^ cells/150 µL of PANC-1 cells were subcutaneously injected into the flank of Crlj:SHO-Prkdc^scid^Hr^hr^ female mice (7 weeks old; Charles River Laboratories). KS-58 was dissolved in DMSO and then tenfold diluted by saline. The peptide solution was injected intravenously to the mice bearing tumors once every 2 days for 4 weeks (40 mg/kg, n = 10). The tumor growth rate was recorded every 3 days by measuring the major and minor axes of the tumors formed with a digital caliper. Measurements were transformed into tumor volume using the formula: tumor volume (mm^3^) = major axis × minor axis^2^ × 0.5.

To establish orthotropic xenograft tumor models, 2.5 × 10^6^ cells/50 µL of PANC-1 cells were injected into the pancreas of BALB/cAJcl-nu/nu male mice (8 weeks old; CLEA Japan, Inc.). KS-58 was dissolved in DMSO and then tenfold diluted by saline. The peptide solution was then injected intravenously to the mice bearing tumors once every 2 days for 4 weeks (40 mg/kg, n = 7). Organ weights of the pancreas, liver, and kidney were measured.

In combination administration test, gemcitabine hydrochloride solution (Nipponkayaku) was tenfold diluted by saline and then injected intraperitoneally to the mice bearing tumors once every 4 days for 4 weeks (40 mg/kg, n = 10), and peptide solution prepared as a same manner described above was injected intravenously to the mice bearing tumors once every 2 days for 4 weeks (10, 20, or 40 mg/kg, n = 10). Organ weights of the pancreas were measured.

### MD simulations of K-Ras(G12D)^GDP/GTP^-binding modes of KS-58

MD simulations were carried out using KRpep-2d/K-Ras(G12D)^GDP^ complex structure (PDB ID: 5XCO) as a template. First, KRpep-2d of the complex was computationally substituted with KS-58, GTP and Mg ion to create the structure model of KS-58/K-Ras(G12D)^GDP^ complex and KS-58/K-Ras(G12D)^GTP^ complex using the Builder module in Maestro (Schrödinger, LLC). The simulation was performed using the gDesmond^36^ ver. 5.7 with the OPLS3e force field^37^. The initial model structure was refined using the Protein Preparation Wizard in Maestro and placed into SPC water molecules solvated with 0.15 M NaCl. After minimization and relaxation of the model, the production MD phase was performed for three independent 200 ns simulations with different initial velocities in an isothermal-isobaric (NPT) ensemble at 300 K and 1 bar using a Nose–Hoover thermostat. Long-range electrostatic interactions were computed using the Smooth Particle Mesh Ewald method. All system setups were performed using Maestro. Trajectory coordinates were recorded every 10 ps. The obtained trajectory was processed utilizing the Simulation Event Analysis module in Maestro for the calculations of protein and peptide RMSDs. Binding free energy of peptides on K-Ras(G12D)^GDP/GTP^ was calculated using MM-GBSA (Schrödinger, LLC) from last 50 ns of stable production run. Procedure of the MD simulations and binding free energy calculation of other peptides was identical to KS-58.

### MD simulations of lipid membrane accessibility of peptides

Lipid membrane accessibility of peptides was examined by three independent, 100 ns meta-dynamics (MetaD) simulations using gDesmond version 5.7 (Schrödinger LLC). MetaD simulation has been widely used and enhanced sampling method that allows the sampling of free energy landscapes. In this simulation, we defined the biasing collective variables (CVs) as the distance between the center of mass of the peptide molecule and the center of the mass of lipid layer on the intracellular side. The initial Gaussian hill height was set at 30. The OPLS3e force field was used for the simulations. A large POPC bilayer and SPC water molecules were solvated with 0.15 M NaCl. Peptide molecules were then placed at a random location in the extracellular solvent region, more than 30 Å from the center of mass of the POPC bilayer. The initial internal conformation of peptide was based on the bound conformation of the K-Ras(G12D)^GDP^. After minimization and relaxation of the model, the production MD phase was performed for ten independent 100 ns simulations in the isothermal–isobaric (NPT) ensemble at 300 K and 1 bar using a Nose–Hoover thermostat. The long-range electrostatic interactions were computed using the Smooth Particle Mesh Ewald method. All system setups were performed using Maestro (Schrödinger LLC).

## Supplementary information

Supplementary Information.

## Data Availability

All data of this study are contained within this manuscript.

## Abbreviations

K-Ras: V-Ki-ras2 Kirsten rat sarcoma viral oncogene homolog
N-Ras: Neuroblastoma ras oncogene homolog
H-Ras: Harvey rat sarcoma viral oncogene homolog
GDP: Guanosine diphosphate
GTP: Guanosine triphosphate
GEF: Guanine nucleotide exchange factor
SOS1: Son of sevenless homolog 1
BRAF: B-Raf proto-oncogene serine/threonine kinase
PI3K: Phosphoinositide 3-kinase
GAP: GTPase accelerating protein
BSA: Bovine serum albumin
RP-HPLC: Reverse-phase high performance liquid chromatography
HRP: Horseradish peroxidase
SA: Streptavidin
DTT: Dithiothreitol
DIE: 1,2-Diiodoethane
DIP: 1,3-Diiodopropane
DIB: 1,4-Diiodobutane
Arg: l-Arginine
Cys: l-Cysteine
Pro: l-Proline
Leu: l-Leucine
Tyr: l-Tyrosine
Ile: l-Isoleucine
Ser: l-Serine
Asp: l-Aspartic acid
Val: l-Valine
γmL: Gamma-methyl-l-leucine
Nle: l-Norleucine
Ahep: (S)-2-Aminoheptanoic acid
Aoc: (S)-2-Aminooctanoic acid
Anon: (S)-2-Aminononanoic acid
Adec: (S)-2-Aminodecanoic acid
Cprg: l-Cyclopropylglycine
Cbug: l-Cyclobutylglycine
Cpeng: l-Cyclopentylglycine
Chg: l-Cyclohexylglycine
Cha: l-Cyclohexylalanine
Phg: l-Phenylglycine
Phe: l-Phenylalanine
Trp: l-Tryptophan
Dap: (S)-2,3-Diaminopropanoic acid
4fF: 4-Fluoro-l-phenylalanine
4mF: 4-Methyl-l-phenylalanine
4cF: 4-Chloro-l-phenylalanine
4tfmF: 4-Trifluoromethyl-l-phenylalanine
1NaphA: l-1-Naphthylalanine
2NaphA: l-2-Naphthylalanine
Tyr(Ome): *O*-Methyl-l-tyrosine
βAla: Beta-alanine
γAba: Gamma-aminobutyric acid
^*D*^X: D-amino acid X
_c_(XZ): Cyclization between amino acids X and Z
_c_[XZ]: Cyclization between amino acids X and Z
CPP: Cell-penetrating peptide
WT: Wild type
PK: Pharmacokinetics
MW: Molecular weight
MD: Molecular dynamics
bRo5: Beyond Lipinski’s rule of 5
CsA: Cyclosporine A
PPI: Protein–protein interaction
