# Supplementary material for: Generation of KS-58 as the first K-Ras(G12D)-inhibitory peptide presenting anti-cancer activity in vivo
Source: Sci Rep. 2020 Dec 10;10:21671. doi: 10.1038/s41598-020-78712-5 (PMC7730438; doi:10.1038/s41598-020-78712-5)
Supplement: Supplementary file 1 — Supplementary Information. [file 41598_2020_78712_MOESM1_ESM.docx]

**Generation of KS-58 as the first K-Ras(G12D)-inhibitory peptide presenting anti-cancer activity *in vivo***

Kotaro Sakamoto^1^*, Teruaki Masutani^1^, and Takatsugu Hirokawa^2,3,4^

^1^Research & Development depertment, Ichimaru Pharcos Company Limitd, 318-1 Asagi, Motosu, Gifu, 501-0475 Japan

^2^Cellular and Molecular Biotechnology Reseach Institute, National Institute of Advanced Industrial Science and Technology, 2-4-7 Aomi, Koto-ku, Tokyo, 135-0064 Japan

^3^Transborder Medical Research Center, University of Tsukuba, 1-1-1 Tennodai, Tsukuba, 305-8575 Japan

^4^Division of Biomedical Science, University of Tsukuba, 1-1-1 Tennodai, Tsukuba, 305-8575 Japan

*****Corresponding author: Kotaro Sakamoto

Tel: +81-58-320-1017

Fax: +81-58-320-1060

E-mail: [sakamoto-kotaro@ichimaru.co.jp](mailto:sakamoto-kotaro@ichimaru.co.jp), [weidlichk58@gmail.com](mailto:weidlichk58@gmail.com)

**Keywords:** KRpep-2d, K-Ras(G12D), Cyclic peptide, Bicyclization, MD simulations


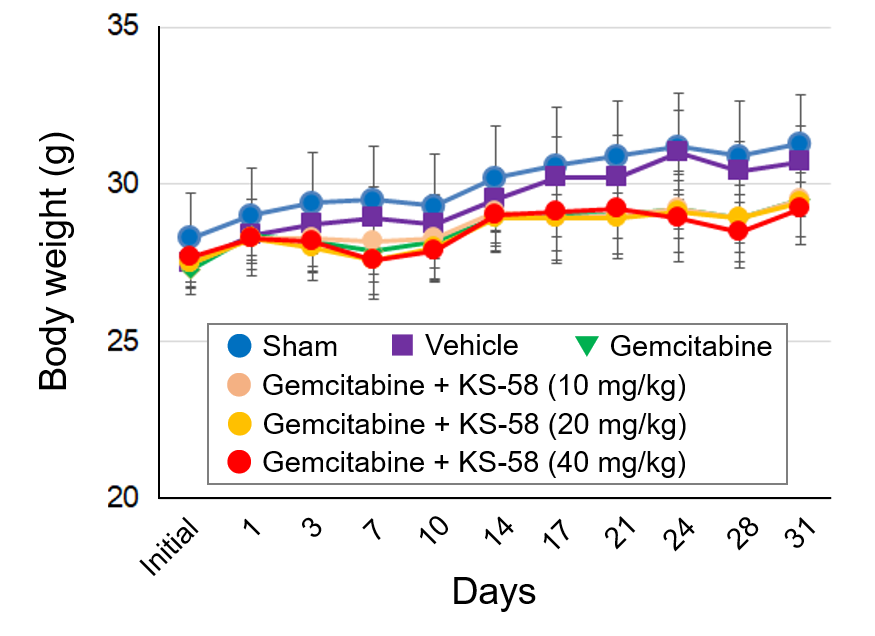


**Supplementary** **Figure** **S1.** Change of body weights of mice in Fig. 5E.


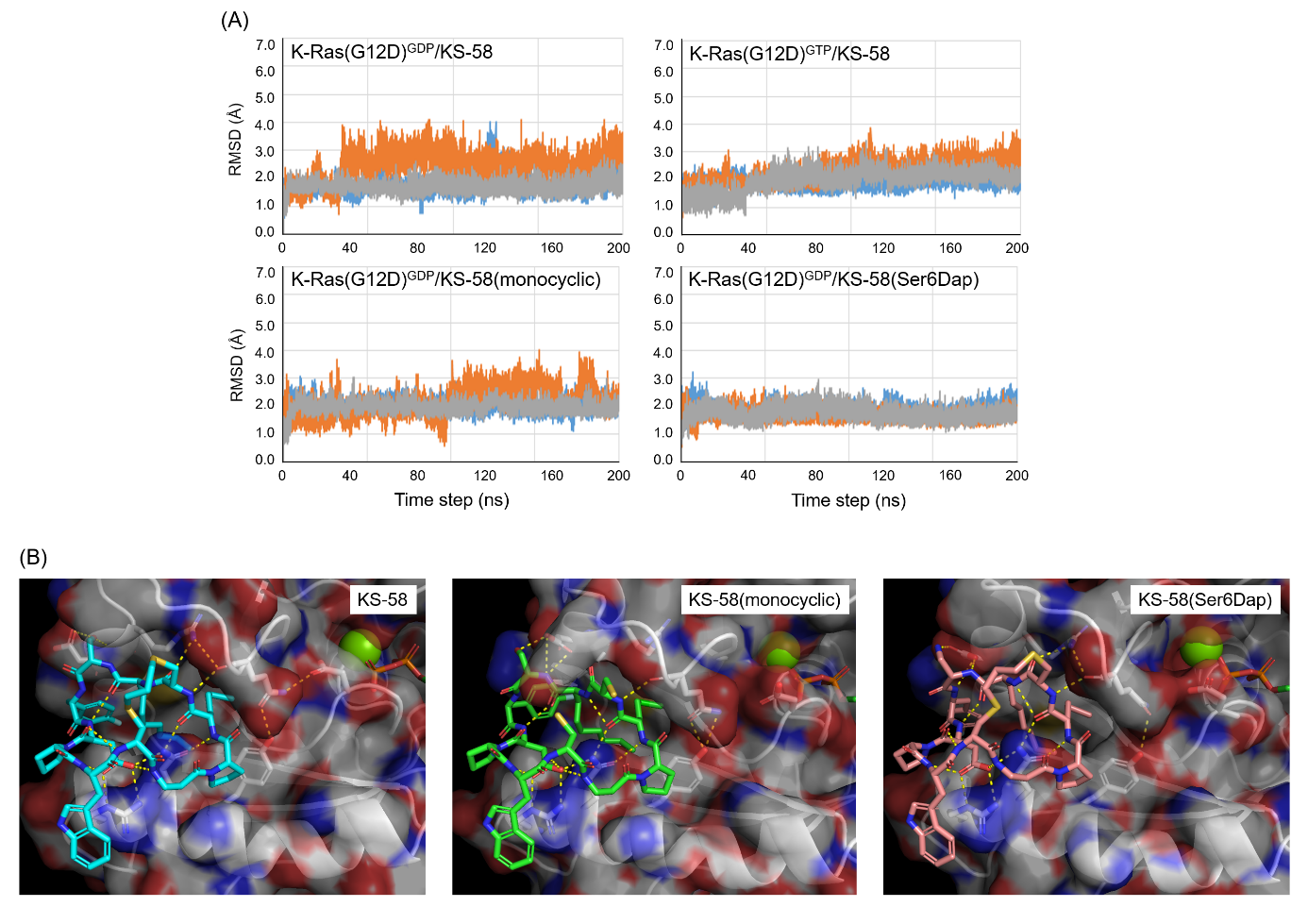


**Supplementary** **Figure** **S2.** (A) The backbone root-mean-square deviations (RMSDs) of KS-58, KS-58(monocyclic), and KS-58(Ser6Dap) from the initial structure after production phase. (B) K-Ras(G12D)^GDP^-binding mode of peptides (stable snap shots). Yellow dash lines indicate hydrogen bonds.


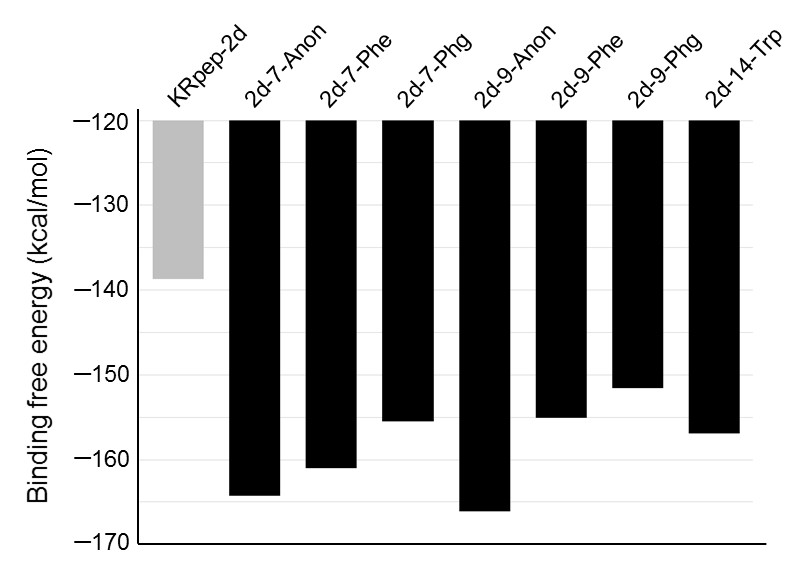


**Supplementary** **Figure** **S3.** Calculated binding free energies of peptides in MD simulations. The bar graph shows the average values of three independent simulations. Peptide name is basically given as 2d-X-Z, wherein X means substituted amino acid position in KRpep-2d and Z means introduced amino acid.

**Supplementary** **Table** **S1.** Analytical data of peptides in this report.

RP-HPLC Mass (Linear mode) (g/mol)

Name T_ret_ (min) Purity (%) Calc. Obsv.^Voltage polarity^

KRpep-2d ^A^8.156 100.00 2561.0 2562.555^POS^

2d-7-γmL ^A^8.231 97.69 2575.0 2575.044^POS^

2d-7-Nle ^A^8.198 97.72 2561.0 2560.518^NEG^

2d-7-Ahep ^A^8.644 100.00 2575.1 2576.787^POS^

2d-7-Aoc ^A^9.176 100.00 2589.1 2590.892^POS^

2d-7-Anon ^A^9.770 98.58 2603.1 2604.907^POS^

2d-7-Adec ^A^10.352 98.53 2617.1 2618.686^POS^

2d-7-Cprg ^A^7.765 100.00 2545.0 2546.403^POS^

2d-7-Cbug ^A^7.936 100.00 2559.0 2560.567^POS^

2d-7-Cpeng ^A^8.159 100.00 2573.0 2573.707^POS^

2d-7-Chg ^A^8.554 100.00 2587.0 2587.472^POS^

2d-7-Phg ^A^8.125 100.00 2581.0 2581.390^POS^

2d-8-Trp ^A^9.174 99.74 2584.1 2585.565^POS^

2d-9-Nle ^A^8.592 100.00 2561.0 2561.200^POS^

2d-9-Ahep ^A^8.943 98.60 2575.1 2574.149^NEG^

2d-9-Aoc ^A^9.402 97.96 2589.1 2588.064^NEG^

2d-9-Anon ^A^9.899 97.85 2603.1 2602.0.32^NEG^

2d-9-Adec ^A^10.412 97.97 2617.1 2616.222^NEG^

2d-9-Cprg ^A^7.643 100.00 2545.0 2546.378^POS^

2d-9-Cbug ^A^8.061 98.91 2559.0 2558.242^NEG^

2d-9-Cpeng ^A^8.194 98.11 2573.1 2572.194^NEG^

2d-9-Chg ^A^8.613 98.51 2587.1 2586.326^NEG^

2d-9-Phg ^A^8.127 95.84 2581.0 2580.757^NEG^

2d-10-Dap ^A^7.472 100.00 2560.2 2561.751^POS^

2d-11-4fF ^A^9.455 98.84 2563.0 2563.911^POS^

2d-11-4mF ^A^9.753 99.30 2559.0 2559.853^POS^

2d-11-4cF ^A^9.952 100.00 2579.5 2580.645^POS^

2d-11-4tfmF ^A^10.456 95.29 2613.0 2614.896^POS^

2d-11-Trp ^A^9.555 100.00 2584.1 2585.756^POS^

2d-11-2NaphA ^A^10.125 96.65 2595.1 2594.144^NEG^

2d-11-Cha ^A^10.000 100.00 2551.1 2552.588^POS^

2d-14-Phe ^A^8.668 99.05 2609.1 2610.981^POS^

2d-14-Trp ^A^8.607 95.65 2648.1 2649.935^POS^

2d-14-1NaphA ^A^9.196 99.56 2659.1 2658.177^NEG^

2d-amide ^D^15.649 99.21 2539.9 2538.909^NEG^

2d-nc ^A^8.933 98.42 2502.0 2501.118^NEG^

KS-36 ^A^12.350 99.26 2622.1 2623.832^POS^

MC-βAla/Cys ^C^10.407 100.00 1164.4 1164.052^POS^

BC-βAla/Cys/DIE ^C^9.910 100.00 1190.4 1189.976^POS^

BC-βAla/Cys/DIP ^C^10.328 96.72 1204.4 1204.490^POS^

BC-βAla/Cys/DIB ^C^10.721 97.64 1218.5 1217.871^NEG^

MC-βAla/*^D^*Cys ^C^10.443 100.00 1164.4 1164.208^POS^

BC-βAla/*^D^*Cys/DIE ^C^9.912 95.43 1190.4 1189.976^POS^

BC-βAla/*^D^*Cys/DIP ^C^10.363 99.04 1204.4 1204.403^POS^

BC-βAla/*^D^*Cys/DIB ^C^10.407 100.00 1218.5 1218.464^POS^

MC-γAba/Cys ^C^10.704 100.00 1178.4 1178.081^POS^

BC-γAba/Cys/DIE ^C^9.931 100.00 1204.4 1204.035^POS^

BC-γAba/Cys/DIP ^C^10.812 97.90 1218.4 1218.376^POS^

BC-γAba/Cys/DIB ^C^11.055 100.00 1232.5 1232.544^POS^

MC-γAba/*^D^*Cys ^C^10.785 100.00 1178.4 1178.170^POS^

BC-γAba/*^D^*Cys/DIE ^C^10.026 100.00 1204.4 1204.209^POS^

BC-γAba/*^D^*Cys/DIP ^C^10.730 99.14 1218.4 1217.863^POS^

BC-γAba/*^D^*Cys/DIB ^C^11.370 100.00 1232.5 1232.601^POS^

BC-βAla/*^D^*Cys/DIP(Anon^5^) ^C^13.079 99.05 1246.5 1245.477^NEG^

BC-βAla/*^D^*Cys/DIP(Anon^5^/Phe^10^) ^C^14.212 99.04 1294.5 1294.114^NEG^

BC-βAla/*^D^*Cys/DIP(Anon^5^/1NaphA^10^) ^C^15.397 98.88 1344.5 1344.266^POS^

BC-βAla/*^D^*Cys/DIP(Anon^5^/Trp^10^) = KS-58 ^C^13.787 95.32 1333.6 1333.620^POS^

BC-βAla/*^D^*Cys/DIP(Adec^5^/Trp^10^) ^C^14.790 98.88 1347.6 1347.519^NEG^

BC-βAla/*^D^*Cys/DIP(Anon^3^/Trp^10^) ^C^13.800 97.27 1333.5 1333.411^NEG^

BC-βAla/*^D^*Cys/DIP(Adec^3^/Trp^10^) ^C^14.754 97.85 1347.6 1347.354^NEG^

BC-βAla/*^D^*Cys/DIP(Aoc^3^/Aoc^5^/Trp^10^) ^C^14.587 98.75 1347.6 1347.569^NEG^

BC-βAla/*^D^*Cys/DIP(Anon^3^/Anon^5^/Trp^10^) ^C^16.421 95.03 1375.6 1375.445^POS^

KS-58(monocyclic) ^C^14.177 95.54 1293.6 1293.307^POS^

KS-58(4fF7TyrOme) ^C^13.577 99.16 1345.6 1345.871^NEG^

KS-58(4fF7Trp) ^C^13.298 99.19 1354.6 1354.677^NEG^

KS-58(4fF7Cha) ^C^15.450 100.00 1321.6 1321.630^NEG^

KS-58(Ser6Dap) ^C^10.092 100.00 1332.7 1332.330^POS^

Biotin-KRpep-2d ^A^8.278 97.06 2745.2 2745.495^POS^

Biotin-KS-58 ^C^12.295 100.00 1704.1 1704.863^POS^

Biotin-KS-58(monocyclic) ^C^12.652 100.00 1664.0 1663.683^NEG^

Peptide name is basically given as 2d-X-Z, wherein X means substituted amino acid position in KRpep-2d and Z means introduced amino acid. Retention times and purities of peptides were characterized by RP-HPLC using SunFire C18 5 µm column (4.6 × 150 mm), at wavelength of 220 nm, under linear gradient four conditions: (A) 10−60% acetonitrile; (B) 5−40% acetonitrile; or (C) 20−90% acetonitrile in water containing 0.1% TFA for 20 min (1 mL/min); or (D) 5−65% acetonitrile in water containing 0.1% TFA for 30 min (1 mL/min). Molecular weights of peptides were determined by the autoflex speed MALDI-TOF mass spectrometer.
